# Nonradioactive heteroduplex tracking assay for the detection of minority-variant chloroquine-resistant *Plasmodium falciparum *in Madagascar

**DOI:** 10.1186/1475-2875-8-47

**Published:** 2009-03-16

**Authors:** Jonathan J Juliano, Milijaona Randrianarivelojosia, Benjamin Ramarosandratana, Frédéric Ariey, Victor Mwapasa, Steven R Meshnick

**Affiliations:** 1 Division of Infectious Diseases, University of North Carolina, School of Medicine, Chapel Hill, NC, USA; 2 Unité de Recherche sur le Paludisme, Institut Pasteur de Madagascar, Antananarivo, Madagascar; 3 Service de Lutte contre le Paludisme, Antananarivo, Madagascar; 4 Institut Pasteur du Cambodge, Phnom Penh, Cambodia; 5 Department of Community Health, College of Medicine, University of Malawi, Blantyre, Malawi; 6 Department of Epidemiology, University of North Carolina, Gillings School of Global Public Health, Chapel Hill, NC, USA

## Abstract

**Background:**

Strains of *Plasmodium falciparum *genetically resistant to chloroquine (CQ) due to the presence of *pfcrt *76T appear to have been recently introduced to the island of Madagascar. The prevalence of such resistant genotypes is reported to be low (< 3%) when evaluated by conventional PCR. However, these methods are insensitive to low levels of mutant parasites present in patients with polyclonal infections. Thus, the current estimates may be an under representation of the prevalence of the CQ-resistant *P. falciparum *isolates on the island. Previously, minority variant chloroquine resistant parasites were described in Malawian patients using an isotopic heteroduplex tracking assay (HTA), which can detect *pfcrt *76T-bearing *P. falciparum *minority variants in individual patients that were undetectable by conventional PCR. However, as this assay required a radiolabeled probe, it could not be used in many resource-limited settings.

**Methods:**

This study describes a digoxigenin (DIG)-labeled chemiluminescent heteroduplex tracking assay (DIG-HTA) to detect *pfcrt *76T-bearing minority variant *P. falciparum*. This assay was compared to restriction fragment length polymorphism (RFLP) analysis and to the isotopic HTA for detection of genetically CQ-resistant parasites in clinical samples.

**Results:**

Thirty one clinical *P. falciparum *isolates (15 primary isolates and 16 recurrent isolates) from 17 Malagasy children treated with CQ for uncomplicated malaria were genotyped for the *pfcrt *K76T mutation. Two (11.7%) of 17 patients harboured genetically CQ-resistant *P. falciparum *strains after therapy as detected by HTA. RFLP analysis failed to detect any *pfcrt *K76T-bearing isolates.

**Conclusion:**

These findings indicate that genetically CQ-resistant *P. falciparum *are more common than previously thought in Madagascar even though the fitness of the minority variant *pfcrt *76T parasites remains unclear. In addition, HTAs for malaria drug resistance alleles are promising tools for the surveillance of anti-malarial resistance. The use of a non-radioactive label allows for the use of HTAs in malaria endemic countries.

## Background

Drug-resistant *Plasmodium falciparum *malaria is a major global public health challenge since anti-malarial use plays a pivotal role for malaria control [1,2]. Identification of molecular markers associated with drug resistance and the development of molecular methods to detect these mutations in parasite samples from patients has allowed for the use of large scale population surveys to infer the efficacy of anti-malarials [3]. In addition, surveillance based on these markers is being implemented to guide national anti-malarial policies in many countries. However, strong associations between molecular markers and clinical outcomes have not been found systematically [1,4].

Currently used techniques and protocols for monitoring anti-malarial resistance based on molecular markers may be inadequate. These methods may fail to detect the emergence of drug resistance mutations in a population due to a lack of sensitivity that is inherent in the method [5-7]. Among the chief reasons for this is the polyclonality of malaria infections in highly endemic areas [1,5,8,9]. Each patient with a polyclonal infection harbours multiple different variants, some of which will represent minority populations within that individual (< 20% of the total parasite population). The term "minority variant" has been used to describe these populations, whether drug resistant or not, in other mixed population infections, such as HIV. Most currently used malaria genotyping methods are insensitive to small sub-populations of drug-resistant parasites (drug-resistant minority variants) in mixed infections [5,7]. Thus, conventional genotyping methods may under estimate the true prevalence of a mutation in a population by missing patients who harbour minority variant drug-resistant parasites and, therefore, delay appropriate anti-malarial policy changes.

In contrast, heteroduplex tracking assays (HTAs), which rely on gel mobility shifts to detect single nucleotide polymorphisms (SNP), are sensitive to minority variants and are quantitative for the relative frequencies of resistant and sensitive parasite variants in a patient. These assays have been used frequently to describe drug-resistant minority variants in HIV. In addition, they have been used to describe drug-resistant minority variant malaria in Malawi and to estimate the complexity of *P. falciparum *malaria infections [5,6,8-10]. Thus, the use of HTAs for monitoring drug resistance mutations in *P. falciparum *may provide a more accurate evaluation of resistance. However, current HTAs rely on isotopic labels for detection of the heteroduplexes, which makes them impractical in most resource-poor settings. In order for them to be used widely, non-isotopic HTA probes are needed.

Previously, an isotopic multi-site-specific HTA (MSS-HTA) was used to describe the presence of minority variant chloroquine (CQ) resistant parasites in patients from Malawi, where the prevalence of the *pfcrt *76T mutation has decreased dramatically since the withdrawal of CQ [5]. Minority-variant *pfcrt *76T bearing parasites were common even though, after the withdrawal of chloroquine, the mutation was undetectable by standard PCR. However, no one has ever looked for minority variants while drug resistance is newly emerging. Unlike Malawi, CQ has been used on the island of Madagascar since 1945 and remains a commonly used anti-malarial for in-home treatment of fever [11]. Despite this fact, CQ-resistant *P. falciparum *strains harbouring *pfcrt *76T were not detected on the island until recently [11]. This may have been due to the geographic isolation of the country [12]. However there is significant concern about the continued introduction of drug resistant parasites from surrounding islands to Madagascar. A recent survey of passengers arriving at the seaport and airport of Mahajanga City, the location of most travel between Madagascar and the Comoros, found high levels of *pfcrt *76T (80.1%), *dhfr *108N (95.0%) and *pfmdr-1 *86Y (99.3%) in passengers arriving with PCR detected malaria [12]. Interestingly, the clinical efficacy of CQ in Madagascar has declined despite the lack of the *pfcrt *76T mutation among the parasites, with efficacy rates as low as 66% [13]. Thus, the epidemiology of CQ resistant malaria in Madagascar provides a unique opportunity to evaluate the use of HTAs to study the emergence of a known drug resistance mutation.

In this paper, a new digoxigenin (DIG)-labeled heteroduplex tracking assay (DIG-HTA) is described, which uses chemiluminescence or chemifluorescence, for the detection of the *pfcrt *76T mutation. This represents the initial development of HTAs appropriate for widespread use in anti-malarial resistance surveillance, in an attempt to track the emergence of mutant *pfcrt *bearing parasites in Madagascar. Like the MSS-HTA, this assay is multi-site-specific and can distinguish the two major CQ resistant haplotypes of *P. falciparum *[5]. The DIG-HTA was compared to the MSS-HTA in clinical samples previously assayed from Malawi to determine the sensitivity and specificity of the assay to detect minority variants. The DIG-HTA, MSS-HTA, and restriction fragment length polymorphism (RFLP) analysis were then used to evaluate clinical samples from Malagasy children who took part in CQ therapeutic efficacy trials as part of the activities of the Réseau d'Etude de la Résistance du Paludisme (RER- a national network for the surveillance of malaria resistance in Madagascar) [14].

## Methods

### Malaria genomic DNA stocks

*Plasmodium falciparum *genomic DNA used for the development of the DIG-HTA was supplied by MR-4 (ATCC, Manassas, VA). The *P. falciparum *strain 3D7 (MRA-102G) was used for wild type DNA. Two mutant *pfcrt *strains were used: K1 (MRA-159G) and 7G8 (MRA-152G). Strain K1 contains the CVIET resistant haplotype, based on the amino acid sequence from codon 72 to codon 76, while strain 7G8 contains the SVMNT resistant haplotype.

### Generation of the DIG-labeled HTA probe

The methods for the probe generation, including site directed mutagenesis, probe screening and insertion into the pT7 Blue vector (Novagen, Inc., Madison, WI), have been described previously [5]. A single stand of the probe was then labeled with a DIG-labeled nucleotide using Klenow fragment. Briefly, the *pfcrt*-pT7 Blue probe-vector construct was harvested using the Hispeed Plasmid Maxi Kit (Qiagen, Valencia, CA). Harvested plasmid was stored at -20°C until use. Ten micrograms of pfcrt-pT7 Blue plasmid construct was digested for one hour with BamHI (New England Biolabs, Beverly, MA) at 37°C and end-labeled in the same buffer by filling in the overhang using 5 μl of labeling master mix incubated at room temperature for 15 minutes [50 μM DIG-dUTP (Roche, Mannheim, Germany), 50 μM dGTP (Promega, Madison, WI), 50 μM dCTP, 50 μM dATP, 10 mM dithiothreitol, and 5 units of Klenow fragment (New England Biolabs)]. The reaction was halted by heat inactivation at 75°C for 15 minutes. The DIG-labeled probe was then cleaved from the plasmid construct by digestion with PstI for 1 hour at 37°C. The labeled probe was then separated from the vector by agarose gel electrophoresis and purified with the QIAquick gel extraction kit (Qiagen). The labeled probe was stored at 4°C until use.

### Heteroduplex tracking assay

The DIG-HTA was performed under the conditions noted by Ngrenngarmlert *et al *with some modifications [9]. PCR amplification of malaria DNA was carried out using primers and conditions already described [5]. An annealing reaction consisting of 8 μl of PCR product (control or clinical sample), 1 μl of 10× annealing buffer (1 M NaCl, 100 mM Tris-HCL, pH 7.5, 20 mM EDTA), 2 μl of 6× loading dye, 0.3 μl 100 pmol CRT HTA F primer, 0.3 μl 100 pmol CRT HTA R primer, and 0.4 μl of DIG-labeled probe was heated to 95°C for 4 minutes and cooled at room temperature for 5 minutes. The annealed heteroduplexes were then separated on a non-denaturing 20% polyacrylamide minigel (Invitrogen, Carlsbad, CA) in 1% Tris-borate-EDTA (TBE) buffer using an Xcell Sure Lock Mini Cell (Invitrogen). All gels were run at a constant current of 17 mA for 2.5 hours per gel. The following controls were used on all gels: water, a non-template control PCR reaction, and PCR reactions from wild type and mutant DNA stocks. The DNA heteroduplexes were then transferred to positively charged Nylon membrane with a pore size of 0.45 μm (Invitrogen) using the Xcell II Blot Module (Invitrogen) in 0.5% TBE buffer. Electroblotting was done at constant voltage (30 V per gel) for 1 hour. Heteroduplexes were fixed to the membrane using an Ultra Lum UV crosslinker (Ultra Lum, Inc., Claremont, CA) at 120,000 μJoules/cm^2^.

### Chemiluminescent and chemifluorescent detection of heteroduplexes

As the heteroduplexes were already labeled with a DIG-labeled probe, no probing step is needed for the membrane. Chemiluminescent detection of the heteroduplexes was carried out per the protocol of the Roche DIG Luminescent Detection Kit (Roche). After addition of the chemiluminescent substrate, disodium 3-(4-methoxyspiro {1,2-dioxetane-3,2'-(5'-chloro)tricyclo [3.3.1.1^3,7^]decan}-4-yl)phenyl phosphate (CSPD), membranes were incubated at 37°C for 5 minutes to enhance the chemiluminescent reaction. For autoradiograhic detection, the membrane was exposed to Hyperfilm ECL (GE Health care, Ltd., Buckinghamshire, UK) for 10 minutes and then developed. For the development of the assay, some chemiluminescent gels were also evaluated using a LAS-3000 imaging system (Fujifilm, Cypress, CA). Images were captured at 10 minute exposures and were processed using Multi Gauge v3.1 software (Fujifilm).

The assay was also evaluated for chemifluorescent detection. The protocol for chemiluminescence was followed until the addition of the CSPD substrate. Instead, ECF substrate for Western Blotting (GE Healthcare) was added to the development folder using 24 μl/cm^2 ^membrane. The membranes were incubated for 5 minutes at room temperature and were imaged using a Molecular Dynamics Storm 860 (GE Healthcare) and analysed using ImageQuant v5.2 software (GE Healthcare).

### Assay design for minority variant detection

The DIG-HTA was run against mixtures of control DNA in quadruplicate. Differing proportions of wild type genomic DNA and CVIET resistant haplotype genomic DNA were mixed to a final sample concentration of 0.1 ng/μl. The sensitivity of the assay was determined by visual inspection for the presence of bands along the dilution series. If a band was not visible to the eye, or only visible in one of the replicates, it was not counted. The assay was tested for chemiluminescent detection, using both autoradiographs and the LUM-3000, as well as chemifluoresecent detection.

In order to evaluate the accuracy of quantitative analysis of band intensity, the dilution series were evaluated as follows. Autoradiographs of the series detected by chemiluminescence were scanned into .jpeg files. The relative intensity of the two bands in each lane was determined using ImageJ (NIH, Bethesda, MD) as described in the documentation. This dilution series was also evaluated for band intensity after imaging by a LAS-3000 (Fujifilm) using Multi Gauge v3.1 software. The intensity of each band was determined by measuring the AUC-BG/mm^2 ^(AUC: area under the curve, BG: background) and the relative intensity of the two bands was then determined for each lane. The chemifluorescent dilution series was quantified using ImageQuant v5.2 software according to the manufacturer's instructions.

To determine if detection remained linear over multiple concentrations, a serial dilution of stock 3D7 DNA was also evaluated by chemiluminescence. The samples were evaluated at 5 minute, 10 minute and 15 minute exposures and quantified as above. To account for variations in detection between gels, the ratio (intensity of a sample/intensity of 1 ng/μl sample) was plotted relative to the concentration of the sample.

### Determination of sensitivity and specificity for minority variants in clinical samples

Twenty four *P. falciparum *isolates from Malawi initially analysed by a radiolabeled MSS-HTA were re-analysed using the DIG- HTA for the presence of minority variant parasites [5]. These samples were known to contain minority variants and their presence had previously been documented by cloning and sequencing [5]. The samples were collected from pregnant women as part of a pilot randomized open label efficacy study of intermittent preventative treatment in pregnancy (IPTp) at rural health clinics. The diversity of these infections, as well as a description of the cohort, study design and informed consent, has been described elsewhere [5,15]. The DIG-labeled HTA was carried out in duplicate on these samples, using the chemiluminescent method with autoradiographic detection. Samples were considered to contain minority variant parasites if bands corresponding to mutant heteroduplexes were visible to the eye in both replicates.

### Detection of minority variants in clinical samples

Thirty one clinical *P. falciparum *isolates from 17 patients were analysed by the DIG-HTA and MSS-HTA for the presence of minority variant *pfcrt *bearing parasites. The samples were collected from consenting patients involved in the assessment of CQ efficacy as part of RER activities between 2002 and 2004 in Sainte Marie and Tsiroanomandidy, Madagascar – prior to the use of insecticide-treated bed nets. Tsiroanomandidy, in the Midwestern foothills, was characterized by mesoendemic malaria [16]. The island of Sainte Marie on the eastern coast was characterized by hyperendemic malaria [16]. The average age of the patients was 80 months (range: 14 to 311 months), with an average parasitaemia of 15,860 trophozoites/μl of blood (range: 88–128,000). In total, 16 recurrent isolates and 15 initial primary (D0) isolates were examined. The DIG-HTA was carried out as noted above for chemiluminescence with autoradiographic detection. Band intensity was quantified as above. The method for the MSS-HTA has been described elsewhere [5]. Samples containing minority variant parasites were PCR amplified and cloned as described by the Topo TA cloning kit (Invitrogen). One hundred colonies from each sample were screened by colony real time PCR as previously described [5]. All colonies that contained mutant DNA and a selection of colonies containing wild-type DNA were sequenced at the University of North Carolina-Chapel Hill Automated DNA Sequencing Facility.

### RFLP analysis for *pfcrt *K76T

All samples found to contain mutant DNA by HTA, as well as a selection of pure wild type samples, were analysed by a RFLP assay that has been used previously in the literature [17].

## Results

**Figure 1:**
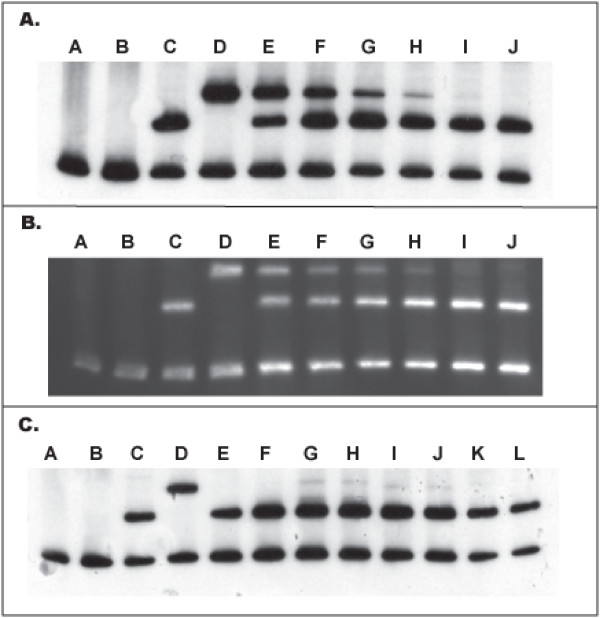
**Digoxigenin(DIG)-labeled heteroduplex tracking assay**. Panel A shows a dilution series of known mixtures of PCR amplified genomic DNA detected by chemiluminescence and autoradiography. Lanes A to D represent controls (water, NTC, Wild type DNA, and Mutant DNA). Mixtures of 50%, 20%, 10%, 5%, 1% and 0.1% mutant DNA are seen in lanes E to J, repectively. Panel B shows a similar dilution series detected by chemiluminesence using the LAS-3000. Panel C shows clinical samples tested by chemiluminescence with autoradiography. Lanes A to D are controls in the same order as Panel A. Lanes E, F, K and L contain samples in duplicate that are pure wild type DNA. Lanes G, H, I and J contain samples in duplicate of mixed parasitaemias. The lowest band on all the gels represents the probe homoduplex.

Like the previously described MSS-HTA, the DIG-HTA probe formed heteroduplexes with differing mobilities when bound to wild type *P. falciparum *DNA amplicons (Figure 1, Panel A, Lane C) and to mutant DNA amplicons from the two major resistant haplotypes: SVMNT (data not shown) and CVIET (Figure 1, Panel A, Lane D). The sensitivity of the DIG-HTA for minority variants was determined using artificial mixtures of know quantities of wild type and mutant *P. falciparum *genomic DNA. The assay was tested using three different detection methods: 1) chemiluminescence with autoradiography (Figure 1, Panel A), 2) chemiluminescence with direct detection using a cooled charge coupled device (CCD) camera (Figure 1, Panel B), and 3) chemifluorescence with direct detection using a phosphorimager. The assay was able to reliably detect populations of mutant parasites to a level of 5% of the total population by all three methods (Table 1). At the 1% mutant level, the assay detected the presence of minority variants in three of four replicates by autoradiography and chemifluorescence. This level of mutant parasite DNA was successfully detected in all four replicates using the CCD camera.

**Table 1:** Quantification of band intensity by the Digoxigenin(DIG)-labeled heteroduplex tracking assay

**% Mutant DNA**	**Autoradiography^1^** **(ImageJ)**		**Chemiluminescent** **(Multi Gauge)**		**Chemifluorescent** **(ImageQuant)**	
	
	Average %	SD	Average %	SD	Average %	SD
50%	45.4	2.7	44.3	1.6	43.5	8.7

20%	29.2	2.5	26.0	2.2	16.2	5.0

10%	15.6	7.5	17.5	5.8	8.7	2.4

5%	8.1	2.2	9.0	3.8	5.4	2.2

1%	1.7^2^	2.2	2.9	1.5	0.85^2^	0.72

0.1%	ND^3^	ND	ND	ND	ND	ND

^1^: Chemiluminescent detection.

^2^: Detected in 3 of 4 replicates.

^3^: Not detected

**Figure 2:**
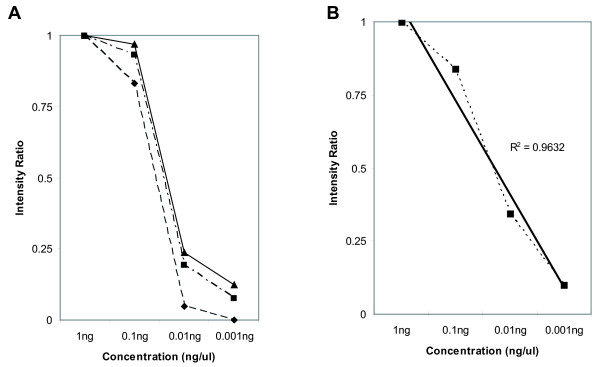
**Dynamic range of imaging modalities**. Panel A shows the detected band intensities by autoradiography of a serial dilution of genomic DNA detected by the DIG-HTA. Intensities are expressed as the ratio of the sample's intensity divided by the intensity of the highest concentration sample in order to account for inter gel variability. The gels were exposed for 5 minutes (diamonds), 10 minutes (squares) and 15 minutes (triangles). Panel B shows the same dilution series detected by CCD camera at 10 minutes (squares) with the best fit trend line (solid black line). The 5 minute and 15 minute detections are not shown.

**Figure 3:**
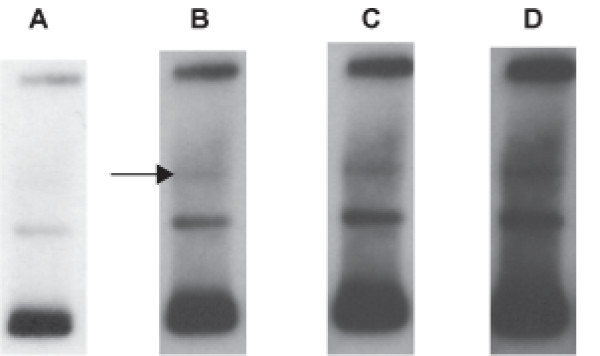
**Saturation of autoradiography**. The figure shows a minority variant mixed sample exposed to autoradiography film for differing intervals. Lane A shows a 5 minute exposure where the mutant band is not visible. Lane B is a 10 minute exposure where the mutant band (see arrow) becomes visible. Lanes C and D show 15 and 20 minute exposures, respectively. In these lanes the background intensity begins to obscure the minority variant mutant band.

The DIG-HTA was also able to reproducibly quantitate the relative amount of DNA in the mixtures of genomic DNA (Table 1). However, each of the detection methods has a different dynamic range of detection. Therefore, different concentrations of control *P. falciparum *DNA were analysed by each method to determine the dynamic range in which quantification remains linear. Figure 2 shows the relative intensity of the detected bands, expressed as a ratio of the individual band intensity over the intensity of the band from the highest concentration. This was done in order to normalize for variability in detection between the gels. Autoradiography (Figure 2, Panel A) has the narrowest dynamic range. The signal for 0.1 ng of genomic DNA becomes nearly saturated at 10 minutes or longer of exposure. However, at 5 minutes of exposure the lower concentrations of DNA are not easily detectable. This dynamic can potentially affect the ability to detect and quantify minority variants (Figure 3). CCD cameras and phosphorimagers have a larger dynamic range and detection remains linear over a larger range. Figure 2 Panel B shows a 10 minute exposure to a CCD camera of a dilution series of DNA detected by chemiluminescence. The relationship remains linear over the entire concentration range (R^2 ^= 0.9832) and over different exposure times [5 minute exposure (R^2 ^= 0.9629) and 15 minute exposure (R^2 ^= 0.9679)].

Of 24 *P. falciparum *isolates from Malawi typed both by MSS-HTA and DIG-HTA, 23 samples were successfully amplified. Eight of the amplified samples contained minority variant drug-resistant parasites detected by MSS-HTA, ranging from approximately 2–5% of the parasite population. The DIG-HTA successfully identified seven of eight samples and no false positive samples were seen (Figure 1, Panel C). Thus, among the samples successfully amplified, the DIG-HTA had a sensitivity of 87.5% and specificity of 100% when compared to the MSS-HTA.

The DIG-HTA and MSS-HTA were then applied to initial and recurrent parasitaemia clinical samples from 17 malaria-positive Malagasy children. The results for each patient are summarized in Table 2. Both assays successfully amplified 30 of 31 available samples. The lack of available DNA prohibited the analysis of the other samples. In total, 2 (6.7%) of 30 samples [CI95%: 0.8–22%] contained CVIET-resistant haplotype *P. falciparum*. Both of these samples (136TDD Day 14 and 373STM Day 14) contained minority variant drug-resistant parasites and were from recurrent parasitaemia samples after initial treatment with chloroquine. The mutant populations represented 1.7% and 2.9% of the recurrent parasite population as detected by DIG-HTA (Table 2). These drug-resistant minority variants were also found by MSS-HTA (2.2% and 1.8%). Neither of these patients harboured detectable CVIET-resistant haplotype parasites prior to treatment. In total, 2 (11.7%) of 17 patients [IC95%: 1.5–36.4%] carried *pfcrt *76T-bearing parasites at some point during their illness. RFLP analyses were unable to detect *pfcrt *76T-bearing parasites in any patient sample.

**Table 2:** Chloroquine resistance DIG-HTA genotyping data of paired Malagasy *Plasmodium falciparum *samples

	**Initial Parasitaemia**			**Recurrent Parasitaemia**			
Patient	Parasitaemia^1^	% Wild Type	% Mutant	Day	Parasitaemia^1^	% Wild Type	% Mutant

36TDD	93250	100	0	14	2000	100	0

48TDD	750	100	0	14	100	100	0

60TDD	250	100	0	14	3500	100	0

62TDD	5000	100	0	14	500	100	0

85TDD	2000	100	0	14	250	100	0

99TDD	11000	100	0	12	5325	100	0

117TDD	750	100	0	12	750	100	0

135TDD	128000	100	0	14	240	DNA^2^	DNA

136TDD	11750	100	0	14	8500	98.3	1.7^4^

121TDD	20000	100	0	-^3^	-	-	-

106STM	-^3^	-	-	14	88	100	0

114STM	-^3^	-	-	14	50000	100	0

233STM	15720	100	0	14	2320	100	0

273STM	1250	100	0	14	1200	100	0

313STM	15720	100	0	14	2320	100	0

315STM	2480	100	0	14	1061	100	0

373STM	54240	100	0	14	1600	97.1	2.9^5^

^1^: Trophozoites per microliter

^2^: Did not amplify.

^3^: No DNA available for sample.

^4^: The MSS-HTA detected 2.2% mutant parasites in this sample. RFLP detected no mutant parasites.

^5^: The MSS-HTA detected 1.8% mutant parasites in this sample. RFLP detected no mutant parasites.

In order to confirm the presence of mutant DNA in the samples, the two clinical samples containing minority variants were cloned and 100 colonies from each were screened by colony real time PCR [5]. Of the screened colonies 1 (1%) from each sample contained mutant DNA. The mutant DNA-plasmid construct was harvested from each of these colonies, as well as 4 wild-type plasmid constructs, and sent for sequencing. Both colonies with detected mutant DNA contained the sequence of the CVIET-resistant haplotype, while none of the wild-type plasmid constructs contained the *pfcrt *K76T mutation.

## Discussion

Until recently, malaria parasites containing mutant *pfcrt *had not been described in Madagascar [11]. The *pfcrt *mutation was reported to occur in 3.3% (6 of 183 samples) of *P. falciparum *isolates from the northern region (Andapa) and from the Midwestern region (Tsiroanomandidy) as detected by RFLP analysis and confirmed by sequencing [11]. In this study, by using HTAs, a higher prevalence of CQ resistant *P. falciparum *was detected, which exist as drug-resistant minority variants in children previously treated with CQ. Among 17 children, 11.7% (CI95%: 1.5 – 36.4%) carried minority variant drug resistant parasites after treatment with CQ monotherapy. RFLP analysis of these same samples failed to detect any containing resistant parasites. Sequence analysis confirmed the presence of minority variant drug-resistant parasites carrying the CVIET haplotype. This haplotype, as well as the CVIDT haplotype, were described upon the initial characterization of *pfcrt *mutations in Madagascar [11].

The findings of this study raise several interesting questions. It appears that genotypically CQ-resistant parasites occur only in a minority of patients in Madagascar. They also occur as a minority population with-in individual patients after therapy with CQ. Despite this, the clinical efficacy of CQ has decreased significantly in Madagascar. This means that the CVMNK CQ-sensitive haplotype is the dominant haplotype detected among CQ failure cases in Madagascar. This suggests one of two possibilities: 1) the patients in this study failed due to other issues, such as pharmacokinetics or 2) there is a *pfcrt *76T independent mechanism causing CQ failure among malaria parasites in Madagascar. All the patients in this study were part of RER clinical efficacy studies and received the WHO recommended 25 mg/kg of CQ base over three days in directly observed therapy. The patients underwent active surveillance for recurrence of parasitaemia (asexual stages) on days 1, 2, 3, 7, etc. These patients were considered clinical treatment failures and did not undergo PCR correction. Further evaluation is required to determine the cause of clinical failures from patients with a dominate population of CVMNK haplotype parasites in the recurrent sample, including detailed pharmacokinetic, human and parasite genetic studies.

In December 2005, the Malagasy national policy for the treatment of uncomplicated malaria was revised abandoning CQ use and implementing artemisinin combination therapy (ACT) with artesunate plus amodiaquine as first-line treatment and artemether plus lumefantrine as second-line treatment. However, CQ remains available commercially (even at the groceries) and pre-packaged CQ is still recommended for in-home treatment of fever (i.e. presumptive malaria cases) among children under five. Even though the fitness of the genetically CQ-resistant minority variant *P. falciparum *remains unclear, the wide-spread presence of the *pfcrt *76T allele could potentially hamper the current policy. The mutation's presence in conjunction with the *pfmdr-1 *86Y mutation has been associated with failure to amodiaquine and previous studies of *pfmdr-1 *86Y in Madagascar have shown a prevalence of 67.5% [16,18]. In addition, as the policy calls for a progressive introduction of ACT, the use of CQ will continue for some time on the island. This will cause further ecological pressure that could potentially promote the spread of the *pfcrt *76T allele. Thus, this finding of the occurrence of minority variant of CQ- resistant *P. falciparum *is potentially helpful by strengthening anti-malarial drug resistance surveillance data. Larger studies would be needed to confirm the distribution of minority variant resistant parasites on the island.

Previously, isotopic HTAs were used to document the presence of minority variant drug resistant parasites in Malawi (*pfcrt *76T and *dhfr *164L) [5,6]. This report describes the first use of a non-isotopic labeled HTA probe to successfully detect minority variant drug resistant *P. falciparum *parasites. This advance will allow this type of method to be used in locations where it was previously prohibited without a significant decrease in the sensitivity of the assay relative to an isotopic label. However, the success of measuring the abundance of DNA by HTA depends on calibrating the signal from the DIG label over a usable range of concentrations [19]. A CCD camera or phosphorimager typically allows signal to be detected in a linear fashion over a larger dynamic range (approximately 10^5^) and sensitivity is generally increased [19]. The DIG-HTA provided band intensities that remained linear when detected by CCD camera over the usual concentration of DNA extracted from filter paper blood spots (Figure 2, Panel B). This becomes an issue when band intensity is determined by autoradiography with densitometry, which typically has a narrow dynamic range (approximately 10^2^) [19]. Film based detection has several disadvantages. At low DNA concentrations, film exhibits reciprocity failure, which means it requires a threshold level of signal before an image is generated [19]. At higher DNA concentrations, saturation effects mean the film darkening is not linear with respect to the amount of light produced [19]. These effects are seen in Figure 2, Panel A. Thus, when using film based detection, it may be necessary to take several exposures to ensure the blot falls within the linear range of the film or adjust the starting amount or concentration of DNA to a specific level. However, in resource poor settings, the use of film-based detection eliminates the need for expensive detection and analysis equipment ($20–60,000 for a CCD camera or phosphorimager).

The use of radioactive probes for HTAs had several major drawbacks. Firstly, disposal of radioactive waste in most developing countries is not possible. Second, as the assays used low energy isotopes (e.g. S^35^), long exposure times ranging from 24 to 48 hours were required to detect a signal. Higher energy isotopes, such as I^125 ^and P^32 ^are more hazardous and require special permits [20]. Lastly, once a probe is labeled, radioactive decay limits the usable life time of the probe [20]. The use of chemiluminescence or chemifluorescence alleviates many of these problems. These techniques are sensitive with fast detection (minutes instead of hours), and are quantifyable. Once probes are bound to the label, they remain relatively stable and do not suffer from decay as quickly as radiolabeled probes [20]. The technology is safe and usable in almost any location.

The International Atomic Energy Agency (IAEA) has supported a number of laboratories in Africa, including Madagascar, to develop isotopic labeling techniques and at these facilities isotopic HTAs would likely be the preferred method [21]. However, these facilities remain relatively uncommon. In order for HTAs to gain wide spread use, alternative labeling techniques are required. The DIG-HTA potentially fulfills this niche. The fact that the label can be detected by X-ray film means that this technique would be easily implemented in a larger number of facilities than would be possible for an isotopic HTA. The use of a CCD camera or phosphorimager will allow for a wider dynamic range to be detected, as described above, but are not required in order to use the assay. It is unlikely that access to these imaging systems will become widespread in the near future.

HTAs appear to be a promising tool for studying the emergence of drug resistance alleles, as well as a useful tool for large scale molecular surveillance of anti-malarial resistance. This is because they address several of the flaws inherent in the currently used molecular methods and genotyping protocols. First, HTAs are more sensitive for minority variants than the currently used nested PCR techniques [5,7]. Therefore they will not underestimate the prevalence of drug resistance alleles in the population and as shown in this study, can detect the emergence of drug resistance alleles in a population earlier than nested PCR [5]. However, the question remains: are drug-resistant minority variants important clinically in an individual host and do they affect drug treatment outcomes? Recent data has shown that *in vitro*, mixed populations of *P. falciparum *containing resistant populations as low as 10% could greatly impact drug treatment outcome [7]. The use of HTAs for genotyping could help us confirm this finding *in vivo *by being implemented in surveillance networks or by evaluating large clinical trials that account for other issues involved in parasite clearance, such as pharmacokinetics and immune function. Second, HTAs can provide a quantitative evaluation of the relative size of sensitive and drug resistant parasite populations in an individual patient. This information can then be used to calculate the relative frequency of drug resistance alleles in the population which can help model the time to drug failure [3,22-24]. This issue has several consequences that were recently reviewed by Hastings and Smith, who have proposed the use of a computer based model (MalHaploFreq) of haplotype frequency as part of anti-malarial resistance surveillance [3]. In addition, the selection coefficient, or fitness, driving the increase in anti-malarial resistance can only be measured by serial evaluation of frequency. The issues of fitness and frequency and their involvement in the spread of anti-malarial resistance were also recently reviewed [3,23,24]. Thus, HTAs allow for direct measurement *in vivo *of the major factor involved in emergence of drug resistance and fitness of specific resistance alleles.

## Conclusion

In conclusion, HTAs appear to be a promising technique for studying the emergence of drug resistance in *P. falciparum *and for use in molecular surveillance of anti-malarial resistance. The advance of using a non-radioactive probe label will allow for these assays to be used in resource limited settings where radioisotopes are prohibited. In this initial study, the biomarker for CQ resistance, *pfcrt *76T was found to be more common than previously reported in Madagascar and exists as minority variants in the population. Thus, the presence of drug-resistant minority variants may be a "marker" for the introduction and emergence of new resistance alleles in a population when parasite frequency is still extremely low in the population. In addition, the ability of HTAs to quantitate parasite populations would allow for evaluation of parasite frequency in the population and more accurate calculations of the spread of drug resistance. Serial evaluation of mutant parasite frequency may allow for a more accurate prediction of time to anti-malarial failure, and in an individual patient, allow for estimation *in vivo *of fitness gains or losses of mutations.

In the future, the use of HTAs in molecular surveillance networks, such as RER in Madagascar or the WorldWide Antimalarial Resistance Network (WWARN), could potentially provide a valuable early warning system for detecting the emergence of genotypic resistance to anti-malarials earlier than standard conventional PCR [4,14,25]. Further work is needed in developing assays for other important resistance loci, evaluation of these techniques in larger studies, and implementing these techniques in the field. In particular, if resistance mutations associated with ACT failure can be identified, HTAs would provide a chance to detect the spread of resistance more quickly and allow for intervention in a timelier manner.

## Abbreviations

HTA: heteroduplex tracking assay; RFLP: restriction fragment length polymorphism; *pfcrt*: *Plasmodium falciparum *chloroquine resistance transporter; CQ: chloroquine; DIG: digoxigenin; *dhfr*: dihydrofolate reductase; *pfmdr-*1: *Plasmodium falciparum *multi-drug resistance gene-1.

## Competing interests

The authors declare that they have no competing interests.

## Authors' contributions

JJJ carried out the assay development, ran all clinical samples and participated in manuscript preparation. MR contributed to experimental design, provided clinical samples and participated in the manuscript preparation. BR and FA contributed to the field work and participated in the preparation of the manuscript. VM contributed to the field work and participated in the preparation of the manuscript. SRM contributed to the experimental design and participated in the manuscript preparation. JJJ, MR and SRM are guarantors of the paper.

## References

[B1] World Health Organization (2005). Susceptibility of *Plasmodium falciparum *to antimalarial drugs. Report on global monitoring: 1996–2004.

[B2] Snow RW, Guerra CA, Noor AM, Myint HY, Hay SI (2005). The global distribution of clinical episodes of *Plasmodium falciparum *malaria. Nature.

[B3] Hastings IM, Smith TA (2008). MalHaploFreq: a computer programme for estimating malaria haplotype frequencies from blood samples. Malar J.

[B4] Plowe CV, Roper C, Barnwell JW, Happi CT, Joshi HH, Mbacham W, Meshnick SR, Mugittu K, Naidoo I, Price RN (2007). World Antimalarial Resistance Network (WARN) III: molecular markers for drug resistant malaria. Malar J.

[B5] Juliano JJ, Kwiek JJ, Cappell K, Mwapasa V, Meshnick SR (2007). Minority-variant pfcrt K76T mutations and chloroquine resistance, Malawi. Emerg Infect Dis.

[B6] Juliano JJ, Trottman P, Mwapasa V, Meshnick SR (2008). Detection of the dihydrofolate reductase-164L mutation in *Plasmodium falciparum *infections from Malawi by heteroduplex tracking assay. Am J Trop Med Hyg.

[B7] Liu S, Mu J, Jiang H, Su XZ (2008). Effects of *Plasmodium falciparum *mixed infections on in vitro antimalarial drug tests and genotyping. Am J Trop Med Hyg.

[B8] Kwiek JJ, Alker AP, Wenink EC, Chaponda M, Kalilani LV, Meshnick SR (2007). Estimating true antimalarial efficacy by heteroduplex tracking assay in patients with complex *Plasmodium falciparum *infections. Antimicrob Agents Chemother.

[B9] Ngrenngarmlert W, Kwiek JJ, Kamwendo DD, Ritola K, Swanstrom R, Wongsrichanalai C, Miller RS, Ittarat W, Meshnick SR (2005). Measuring allelic heterogeneity in *Plasmodium falciparum *by a heteroduplex tracking assay. Am J Trop Med Hyg.

[B10] Delwart EL, Shpaer EG, Louwagie J, McCutchan FE, Grez M, Rubsamen-Waigmann H, Mullins JI (1993). Genetic relationships determined by a DNA heteroduplex mobility assay: analysis of HIV-1 env genes. Science.

[B11] Randrianarivelojosia M, Fidock DA, Belmonte O, Valderramos SG, Mercereau-Puijalon O, Ariey F (2006). First evidence of pfcrt mutant *Plasmodium falciparum *in Madagascar. Trans R Soc Trop Med Hyg.

[B12] Menard D, Randrianarivo-Solofoniaina AE, Ahmed BS, Jahevitra M, Andriantsoanirina V, Rasolofomanana JR, Rabarijaona LP (2007). Drug-resistant malaria parasites introduced into Madagascar from Comoros Islands. Emerg Infect Dis.

[B13] Randrianarivelojosia M, Raveloson A, Randriamanantena A, Juliano JJ, Andrianjafy T, Raharimalala LA, Robert V (2009). Lessons learnt from the six decades of chloroquine use (1945–2005) to control malaria in Madagascar. Trans R Soc Trop Med Hyg.

[B14] Randrianarivelojosia M, Rakotonjanabelo LA, Mauclere P, Ratsimbasoa A, Raharimalala LA, Ariey F (2002). [National Network study to perpetuate the surveillance of *Plasmodium falciparum *sensitivity to antimalarials in Madagascar]. Arch Inst Pasteur Madagascar.

[B15] Kalilani L, Mofolo I, Chaponda M, Rogerson SJ, Alker AP, Kwiek JJ, Meshnick SR (2007). A randomized controlled pilot trial of azithromycin or artesunate added to sulfadoxine-pyrimethamine as treatment for malaria in pregnant women. PLoS ONE.

[B16] Rason MA, Andrianantenaina HB, Ariey F, Raveloson A, Domarle O, Randrianarivelojosia M (2007). Prevalent pfmdr1 n86y mutant *Plasmodium falciparum *in Madagascar despite absence of pfcrt mutant strains. Am J Trop Med Hyg.

[B17] Sarr O, Myrick A, Daily J, Diop BM, Dieng T, Ndir O, Sow PS, Mboup S, Wirth DF (2005). In vivo and in vitro analysis of chloroquine resistance in *Plasmodium falciparum *isolates from Senegal. Parasitol Res.

[B18] Tinto H, Guekoun L, Zongo I, Guiguemde RT, D'Alessandro U, Ouedraogo JB (2008). Chloroquine-resistance molecular markers (Pfcrt T76 and Pfmdr-1 Y86) and amodiaquine resistance in Burkina Faso. Trop Med Int Health.

[B19] Dickinson JaF, SJ, Walker JM (2002). Quantification of Proteins on Western Blots Using ECL. The Protein Protocols Hanbook.

[B20] Campell AK (1988). Chemiluminescence: Principles and Applications in Biology and Medicine.

[B21] IAEA (2008). Detecting drug resistant malaria and tuberculosis in Africa: highlighting acheivements of Regional Technical Cooperation Project RAF/6/025.

[B22] Hartl DL (2000). A Primer of Population Genetics.

[B23] Hastings IM, Donnelly MJ (2005). The impact of antimalarial drug resistance mutations on parasite fitness, and its implications for the evolution of resistance. Drug Resist Updat.

[B24] Mackinnon MJ (2005). Drug resistance models for malaria. Acta Trop.

[B25] Barnes KI, Lindegardh N, Ogundahunsi O, Olliaro P, Plowe CV, Randrianarivelojosia M, Gbotosho GO, Watkins WM, Sibley CH, White NJ (2007). World Antimalarial Resistance Network (WARN) IV: clinical pharmacology. Malar J.

